# Biomimetic poly(amidoamine) hydrogels as synthetic materials for cell culture

**DOI:** 10.1186/1477-3155-6-14

**Published:** 2008-11-17

**Authors:** Emanuela Jacchetti, Elisa Emilitri, Simona Rodighiero, Marco Indrieri, Antonella Gianfelice, Cristina Lenardi, Alessandro Podestà, Elisabetta Ranucci, Paolo Ferruti, Paolo Milani

**Affiliations:** 1 Dipartimento di Fisica, Università di Milano, via Celoria 16, 20133 Milano, Italy; 2 Dipartimento di Chimica Organica e Industriale, Università di Milano, via Venezian 21, 20133 Milano, Italy; 3 Istituto di Fisiologia Generale e Chimica Biologica, Università di Milano, via Trentacoste 2, 20134 Milano, Italy; 4 CIMaINa, Centro Interdisciplinare Materiali e Interfacce Nanostrutturati, Università di Milano, Italy

## Abstract

**Background:**

Poly(amidoamine)s (PAAs) are synthetic polymers endowed with many biologically interesting properties, being highly biocompatible, non toxic and biodegradable. Hydrogels based on PAAs can be easily modified during the synthesis by the introduction of functional co-monomers. Aim of this work is the development and testing of novel amphoteric nanosized poly(amidoamine) hydrogel film incorporating 4-aminobutylguanidine (agmatine) moieties to create RGD-mimicking repeating units for promoting cell adhesion.

**Results:**

A systematic comparative study of the response of an epithelial cell line was performed on hydrogels with agmatine and on non-functionalized amphoteric poly(amidoamine) hydrogels and tissue culture plastic substrates. The cell adhesion on the agmatine containing substrates was comparable to that on plastic substrates and significantly enhanced with respect to the non-functionalized controls. Interestingly, spreading and proliferation on the functionalized supports are slower than on plastic exhibiting the possibility of an easier control of the cell growth kinetics. In order to favor the handling of the samples, a procedure for the production of bi-layered constructs was also developed by means the deposition via spin coating of a thin layer of hydrogel on a pre-treated cover slip.

**Conclusion:**

The obtained results reveal that PAAs hydrogels can be profitably functionalized and, in general, undergo physical and chemical modifications to meet specific requirements. In particular the incorporation of agmatine warrants good potential in the field of cell culturing and the development of supported functionalized hydrogels on cover glass are very promising substrates for applications in cell screening devices.

## Background

In the last years the progress of biological sciences has led to outstanding developments in the field of cell culturing in vitro. Several new techniques, such as cell microarray or cells on chips, require reliable support materials with good biocompatibility and cell adhesion, preferentially disposable and simple to use [1,2]. Among synthetic materials, hydrogels present unique tissue-like properties for interactions with living cells [3,4], such as water content and permeability to oxygen and metabolites. In principle, fully synthetic hydrogels, as opposed to naturally derived media (e.g. gelatin, chitosan, etc.), should be more advantageous, coupling the aforementioned properties with the possibility of complete control over hydrogel composition, cross-linking and swelling. The hydrogels can be produced with tailored shape and thickness, and their surface can be patterned with lithographic techniques [5,6]. Moreover hydrogels can be fittingly functionalized with biomolecules for obtaining customized properties [7]. [8]

Cell adhesion on fully synthetic hydrogels, however, is still an issue for many of these materials, such as PHEMA or crosslinked PEG derivatives [9]. A number of chemical and physical modifications have been proposed to overcome this problem, often relying on modification of the synthetic surface with biological or biomimetic moieties, like peptides or proteins [10]. The process of cell adhesion to a substrate, both on the natural extracellular matrix (ECM) and synthetic materials is mediated by interactions between surface ligands and cell receptors, such as transmembrane integrins and proteoglycans [11]. The tripeptide argininglycin aspartic acid (RGD), present in several ECM proteins, has been object of intensive research in the last years [12]. In fact, several studies have shown that this tripeptide and some of its analogues can interact with adhesion regulating proteins of the integrin family, and play a role in promoting cell adhesion and spreading, mimicking the effect of some ECM proteins such as fibronectin or vitronectin [13-15]. The overall action mechanism is still not completely clear, but some studies have associated it to the conformation of the guanidine side group of arginine and its distance and angle from the acidic pendant of aspartic acid [16,17]. Modification of chemical structures in order to include an RGD or RGD-like group has been proposed for a number of applications where interaction with cells is desired, to enhance adhesion or recognition by cellular receptors [18-20].

Poly(amido amine)s (PAAs) are synthetic polymers highly biocompatible, non toxic and biodegradable [21,22]. Several structures [23,24] including biologic, biomimetic and bioactive compounds, can be incorporated in the PAA network by covalent attachment during the synthesis step [25]. In the hydrogels based on PAAs [26,27] functional co-monomers, as 4-aminobutyl guanidine (agmatine), can be easily introduced in order to build a functional amphoteric repeating unit which is structurally similar to RGD [28]. This new material does not involve peptide synthesis and purification and can be prepared from commercially available materials, with lower costs and a simple one-pot synthesis. Moreover, the versatility of the involved chemistry allows to easily add other functionalities or cell signaling groups that can be inserted during or after the chemical synthesis [23-25].

Ferruti et al. [29] carried out preliminary evaluations of cytotoxicity and cell proliferation on fibroblast cell line as well as of hydrogel degradation tests under conditions mimicking the physiological environment. These pioneering experiments demonstrate that PAA hydrogels containing agmatine are suitable substrate for cell culturing and that the degradation rate depends on the selected aminic cross-linker. The obtained results prompted us to perform a systematic and comparative study on cell adhesion and proliferation between amphoteric agmatine-based PAA hydrogels and not functionalized PAA hydrogels. Moreover, in view of the preparation of inexpensive, disposable and handling devices, a protocol for the preparation of glass supported functional amphoteric PAA hydrogel layers has been developed. A bi-layered construct has been prepared by spin coating a pre-treated glass with this novel functional hydrogel layer, in order to have a stable and functional substrate for cell culture.

In this paper we report our research on cell culture experiments using epithelial MDCK (Madin-Darby canine kidney) cells since they are known to express the RGD-binding *α*_V_*β*_3 _integrin [30]. Cells were plated on glass supported amphoteric PAA-based hydrogels having as control substrate tissue culture plate surfaces (TCPS). Our results indicate that glass supported PAA hydrogels containing agmatine promote cell adhesion and open interesting perspectives for the development of microsystems aimed at realizing increasing cell handling integration on chips.

## Materials

Ethanol, hydrochloric acid (37%), nitric acid (65%), 3-aminopropyltrimethoxy silane, 1,2-diaminoethane (EDA), 4-aminobutylguanidine sulfate (agmatine sulfate) and GRGD peptide were purchased from Sigma-Aldrich and used as received. N,N'-Bis (acrylamido) acetic acid (BAC) was prepared as reported in the literature [31] and purity determined by Nuclear Magnetic Resonance (NMR) and titration; 2-methylpiperazine (Fluka) was recrystallized from heptane. Phosphate buffer solution (PBS) was prepared using Sigma Aldrich tablets (# P4417). One tablet dissolved in 200 ml of deionized water yields 0.01 M phosphate buffer, 0.0027 M potassium chloride and 0.137 M sodium chloride, pH 7.4, at 25°C. Soluble AGMA-1 polymer was prepared as reported in the literature [29]. The sample was characterized by NMR and Gel Permeation Chromatography (GPC). The molecular weight of the sample used: Number average molecular weight = 5500 and Weight average molecular weight = 6500, polydispersity = 1.25; its NMR was consistent with those reported in the literature. TCPS (tissue culture plate surfaces), multiwells, and tissue culture flasks were purchased from Zellkultur und Labortechnologie, Switzerland; round glass coverslips as support for hydrogels (13 mm in diameter, 0.7 mm thickness) from Zeus super. All chemicals used in the biological tests were purchased from Sigma-Aldrich. Sterile and ultrafiltered water, purchased from Fluka (Sigma # 95289), was used during hydrogel synthesis and preparation. From datasheet water is considered endotoxin-free by LAL test. The endotoxin free water was used in the preparation of all cell culture reagents (such as HBSS, PBS, cell culture medium). Since the hydrogels preparation and experiments steps were protected from bacteria contamination, we assume that the final product is over of endotoxin contamination. Spin coating was performed using a Laurell WS-400B-6NPP-Lite spin coater. ^1^H and ^13^C NMR spectra were obtained using a Brüker Avance400 spectrometer operating at 400.132 MHz (1H) and 100.623(13C), and using Brüker software. Size exclusion chromatography (SEC) traces were obtained with Toso-Haas TSK-gel G4000 PW and TSK-gel G3000 PW columns, using a Waters model 515 HPLC pump. The two columns were connected in series and the mobile phase was Tris buffer (pH 8,10); flow rate 1 ml/min; refractive index detector Waters 2410. The samples were prepared in Tris buffer with a 1% concentration in polymer. Molecular weight determinations were based on a pullulan standards calibration curve.

## Methods

### Preparation of the free standing hydrogels

General preparation procedure for AGMA1-75 hydrogel: in a 10 ml round bottomed flask BAC (1099 mg, 5.4 mmol, 97.5%) was added under nitrogen atmosphere and stirring to an aqueous lithium hydroxide solution (lithium hydroxide monohydrate, 226.26 mg 5.4 mmol in 1.8 ml). When the solution was clear, agmatine sulfate (308.17 mg, 1.35 mmol, 97%) and more lithium hydroxide monohydrate (81.9 mg, 2.7 mmol) were added and dissolved. This mixture was allowed to react for 24 hr at room temperature (20 ± 5°C) in the dark, and then EDA (121.7 mg, 2.05 mmol) was added. The solution was stirred for 1 minute, retrieved with a syringe and injected in a square mould made of two silanized 10 × 10 cm^2 ^glass plates separated by a 0.3 mm silicone spacer. The hydrogel was allowed to crosslink at room temperature for 72 hr protected from direct sunlight then it was retrieved as a pliant solid clear film. The PAA hydrogel obtained by this procedure was purified from low molecular weight impurities by first extracting with excess ethanol and then with sterile and ultrafiltered water. Treating directly ethanol-swollen hydrogel samples with aqueous media caused an osmotic shock leading to surface fracture. The adopted procedure was, therefore, to expose the ethanol-swollen hydrogel to water/ethanol mixtures with increasing water concentrations, until pure water was used. The extraction time was at least 30 min for each step. ISA23-75 was prepared and purified using the same procedure, and the following reagents: BAC (1099 mg, 5.4 mmol), lithium hydroxide monohydrate (226.26 mg 5.4 mmol), 2-methylpiperazine (135.3 mg, 1.35 mmol), water (1.8 ml), and EDA (121.7 mg, 2.05 mmol).

### Swelling test

The native (unwashed) hydrogels were cut into 10 × 10 × 0.3 mm^3 ^parallelepiped, weighed (mean weight 206 ± 15 mg), and washed in ethanol/water according to the procedure described above. Each specimen was placed inside a 50 ml beaker containing 30 ml water (or buffer) at the desired temperature. At regular intervals the specimen was taken out of the beaker, any visible surface moisture was wiped off, and then it was weighed. After this, the specimen was returned to the test tube and the uptake of water was measured until the maximum mass was obtained. The percentage amount of water absorbed was calculated using the following formula:

(1) *water*_abs _(%) = (*W*_final _- *W*_dry_)/*W*_dry _× 100,

where *W*_final _and *W*_dry _are the final weight of the swelled hydrogel and the weight of the dry hydrogel respectively. Equilibrium is reached between 5 and 7 hr. After 24 hr, each sample was rinsed in sterile and ultrafiltered water and freeze-dried to obtain the dry weight. Tests were performed in water, PBS, cell culture medium and cell culture medium under culture conditions (37°C, 5% CO_2_).

### Degradation tests

Several samples of dry AGMA1-75 and ISA23-75 were weighed (average weight 18 ± 5 mg) and placed each in a test tube containing 1 ml phosphate buffered solution 0.1 M at pH 7.4. The samples were closed, placed in an incubator at 37°C and retrieved at different times. The recovered samples were blotted dry and weighed, then they were freeze dried to define the dry weight. The proportional swelling was calculated as

(2) *swelling *(%) = *W*_wet_/*W*_initial dry _× 100,

where *W*_wet _is the weight of the swelled hydrogel, *W*_initial dry _is the weight of the initial dry hydrogel. The proportional weight rest is evaluated as

(3) *weight rest *(%) = *W*_final dry_/*W*_initial dry _× 100,

where *W*_final dry _is the weight of the final dry hydrogel.

### Glass amino silane functionalization

Round glass coverslips, 13 mm in diameter, were treated as previously reported [32]. They were soaked in aqua regia at room temperature for 5 hr (20 coverslips were laid out in a glass dish 100 mm in diameter and covered with 12 ml of the acid mixture), washed several times in sterile and ultrafiltered water and then in ethanol before being soaked in a 10% (v/v) ethanol solution of – amino propyltrimethoxy silane (15 ml) overnight. The samples were recovered and washed in ethanol (2 × 20 ml), sterile and ultrafiltered water (3 × 20 ml), and then sonicated in sterile and ultrafiltered water. They were finally dried with soft paper and used within 24 hr.

### Supported hydrogel layer preparation

AGMA1-75: BAC (39 mg 0.197 mmol) was dissolved in sterile and ultrafiltered water (66 *μ*l) together with lithium hydroxide monohydrate (14.5 mg, 0.30 mmol). After the solution cleared, agmatine sulfate (11.20 mg 0.05 mmol) was added and dissolved. The mixture was allowed to react, in the dark and under nitrogen, for 24 hr at room temperature (20 ± 5°C), then EDA (6.4 mg 0.09 mmol) was added just before casting. About 20 *μ*l of the solution were cast on each pre-treated glass coverslip, using a Pasteur pipette, before spin coating them (4 coated glasses are obtained from each preparation). After the deposition, samples were kept in a closed sterile container for 3 days at room temperature, to allow the cross linking reaction to proceed. Then they were retrieved, put each in a well of a multiwell plate and washed as described for the free hydrogels, each sample being soaked in 1 ml solution. 30 min after the last addition of water/ethanol mixture, the solution was removed, and replaced with 1 ml of sterile and ultrafiltered water. Samples were kept in water at 37°C overnight, rinsed in sterile and ultrafiltered water and sterilized with UV-rays for ten minutes before use. ISA23-75: The procedure was the same as reported above for AGMA1-75, using the following quantities: BAC (39 mg, 0.197 mmol) sterile and ultrafiltered water (66 *μ*l), lithium hydroxide monohydrate (8.25 mg, 0.197 mmol), 2-methylpiperazine (5.0 mg 0.05, mmol), EDA (4.2 mg, 0.68 mmol).

### Atomic Force Microscopy

The investigation of morphology of the substrates was carried out in fluid using a Bioscope II AFM (Veeco, USA). The AFM was operated in Tapping Mode at scan rates of 0.4–1.2 Hz over scan areas of 50 × 50 *μ*m^2 ^and 5 × 5 *μ*m^2^. V-shaped silicon nitride cantilevers (DNP-20 SW, Veeco, USA) were used, with resonant frequency in milliQ water ranging from 10 kHz to 20 kHz. The tip holder was cleaned with liquid soap and water before and after each use. The samples were placed inside a glass Petri dish flooded with milliQ water for imaging. AFM images are typically flattened line by line subtracting a polynomial function, in order to get rid of the tilt of the sample and of the scanner bow.

### Cell culture

Immortalized Madin-Darby Canine Kidney epithelial cell line (MDCK) were cultured in Dulbecco's Modified Eagle's Medium, supplemented with 10% Fetal Bovine Serum, 2 mM L-Glutamine, 0.1 mM non essential amminoacid, 1.5 g/l sodium bicarbonate, 1 mM sodium pyruvate, 100 units/ml penicillin and 100 *μ*g/ml streptomycin. Cells were grown in tissue culture flasks at 37°C in controlled atmosphere (5% CO_2_). For cell adhesion and proliferation MDCK cells were seeded at a concentration of 10^4 ^cells/well to 13 mm diameter round glass coverslips coated with AGMA1-75, ISA23-75 and to TCPS.

### Cell adhesion, viability and proliferation

MDCK adhesion on AGMA1-75, ISA23-75 and TCPS were measured. The results were compared in order to evaluate the effectiveness of AGMA1-75 as culture substrate. Cells were monitored every 30 min during the first four hours after cell plating, then every hour for the next 2 or 3 hr. Afterwards they were observed once a day until cells achieved confluence. Images from each sample were collected with a Power Shot G6 Canon digital camera mounted on a Zeiss Axiovert 40 CFL inverted optical microscope using 10× objective lens. Four random fields from each sample were photographed. The number of cells assuming the typical asymmetric morphology (polygonal-like) of adherent MDCK were counted and normalized to the total number of plated cells:

(4) *polygonal-like cell *(%) = *N*_polyg cell_/*N*_tot cell _× 100,

where *N*_polyg cell _is the number of the cells showing the polygonal-like morphology and *N*_tot cell _is the total number of counted cells in each image.

For the cell adhesion experiments in the presence of soluble AGMA1 or GRGD peptide, cells were seeded in culture medium supplemented with 1 mM AGMA1 (calculated on the repeating unit concentration), 10 mM AGMA1 or 1 mM GRGD. After 4 hr the inhibition of adhesion was calculated. It is defined as:

(5) *inhibition *(%) = [1 - (*polygonal-like cell *(%)/*polygonal-like cell *(%)_contr_] × 100,

where *polygonal-like cell *(%) as defined in Expression 4 and *polygonal-like cell *(%)_contr _is the cell adhesion on each substrate (TCPS, AGMA1-75 and ISA23-75) in medium without soluble AGMA1 or GRGD.

Cell viability tests were also carried out for MTT ((3-4,5-dimethylthiazol-2-yl)-2,5-diphenyl tetrazolium bromide (Sigma # M2128)) assay procedure. 4 × 10^4 ^cells were seeded on AGMA1-75 and ISA23-75 hydrogels, and TCPS within a 48-well tissue culture plates. Non-adherent cells were removed by washing at one and three hours from seeding. Then, 500 *μ*l 10% MTT solution (5 mg/ml in PBS) was added to each sample and the plates were incubated for 3 h at 37°C. The supernatant was discarded and the formazan salt was dissolved in an equal volume of acid isopropanol-0.04 M HCl. The absorbance was measured at 570 and 650 nm.

### Immunofluorescence assay

Forty-eight hours after seeding in culture, cells were fixed for 15 min in 3% paraformaldehyde, rinsed with 0.1 M glycine in phosphate buffered saline (PBS) and permeabilized with 0.25% Triton X-100 in PBS for 15 min at room temperature, and processed for direct immunofluorescence analysis: to visualize the distribution and the organization of focal contacts, cells were incubated for 2 hr with 200 *μ*M of FITC (Fluorescein isothiocyanate) mouse anti-vinculin antibody in PBS with 0.1% Tween + 2% BSA, then actin filaments were labeled by 30 min incubation with 1 *μ*g/ml of TRITC (Tetramethylrhodamine isothiocyanate) phalloidin in the same buffer. Nuclei were labeled by 1 *μ*g/ml of DAPI in PBS. Fixed and stained cells were mounted in Mowiol and imaged using confocal microscopy (TCS SP2 AOBS Leica Confocal Microscopy).

## Results

**Figure 1:**
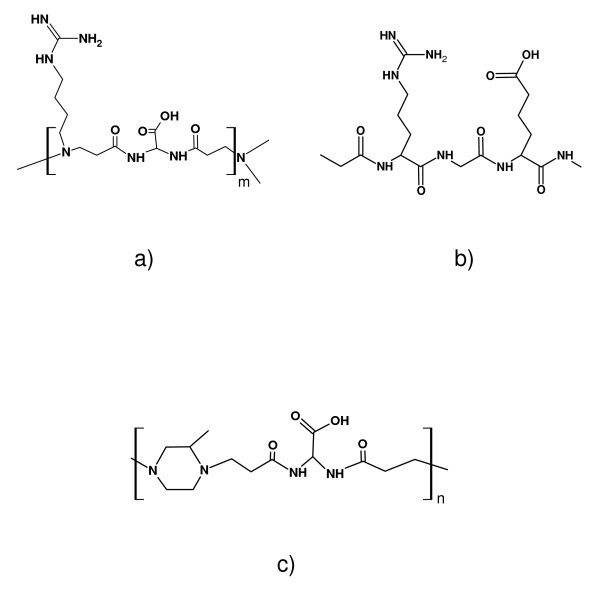
**Repeating units**. Repeating unit of a) AGMA1; b) RGD peptide and c) ISA23.

In this work two hydrogels were tested, namely ISA23 and AGMA1. They are based on different amphoteric PAA structures, both known in the literature as highly biocompatible structures [26-29]. As previously pointed out, the AGMA1 repeating units (Figure 1a)) are very similar to the well known adhesion-modulating RGD peptide sequence (Figure 1b)). Since ISA23 does not carry any guanidine pendant group (Figure 1c)), it is expected do not show any significant cell adhesion properties [33] and it was used as a non-functionalized control. In order to make the hydrogels more handy a new bi-layered system was designed, prepared and tested. It is composed by a functionalized glass support covered with a thin hydrogel layer. The whole system results to be more robust and, at the same time, preserve an optimal optical transparency as required by microscopy characterizations. All the described results were obtained by using these bi-layered constructs as they could represent an interesting approach for addressing effective cell culture and screening devices.

### Swelling properties of hydrogels

**Figure 2:**
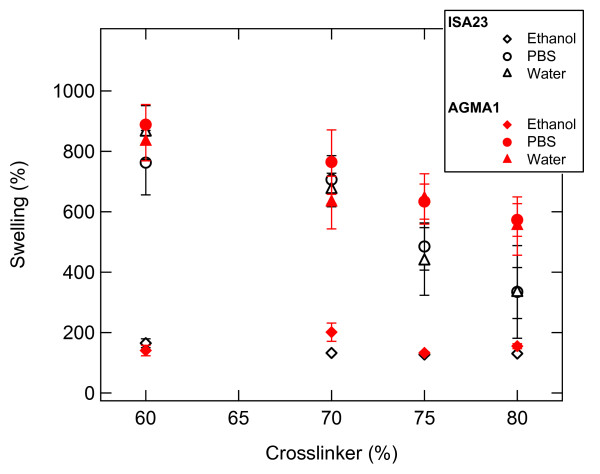
**Swelling**. Swelling (%) versus crosslinker (%) contained in ISA23 and AGMA1 for different fluids.

Among the main parameters that control the swelling rate there are: crosslinking density, network structure and overall hydrophilicity of the polymer chains. Swelling tests for comparing the network of AGMA1 and ISA23 were performed in water, PBS and ethanol. Figure 2 shows the proportional swelling, calculated using Expression 2, versus the percentage of crosslinker contained in each of the two types of hydrogels in the different fluids. At first it can be noted that the amount of absorbed ethanol is the same and constant for both hydrogels independently on the crosslinker amount. In the case of water and PBS the swelling of ISA23 and AGMA1 decreases as a function of the crosslinker content. The amount of water and PBS adsorbed by ISA23 for defined crosslinker content is more less the same (within the experimental error). A similar behaviour can be observed also for AGMA1. However it can be also observed that the swelling for both hydrogels is alike up to a crosslinker amount of 70% after which the two hydrogels begin to show wide apart trends.

### Supported hydrogels

**Figure 3:**
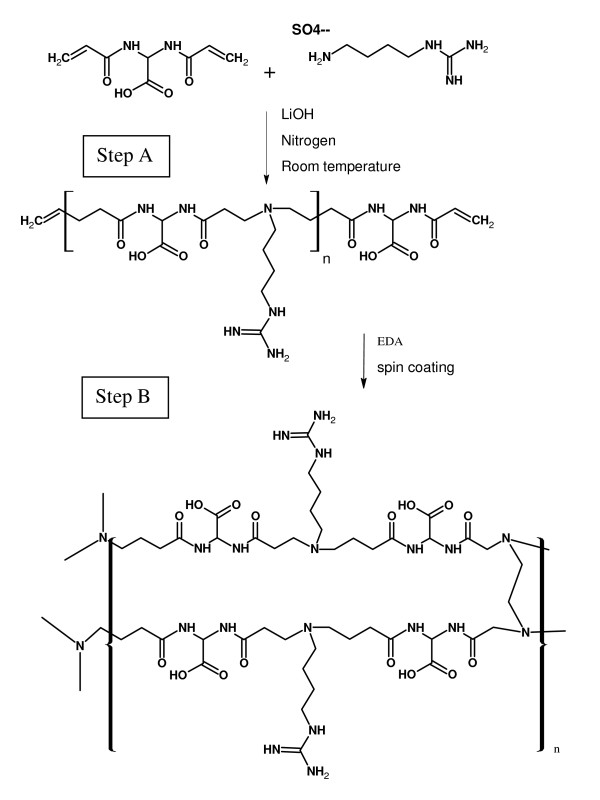
**Synthesis of hydrogels**. Scheme of the procedure for the synthesis of hydrogels. Step A: functional oligomer preparation; step B: in situ crosslinking.

The previously characterized hydrogels were then used for preparing glass supports coated with hydrogels to be used for cell culture. The adhesion between an organic hydrated layer and glass is usually poor, so, in order to prepare a stable construct, the organic layer was "anchored" to the glass using amino silane groups. The hydrogel layer was prepared by a two step synthesis, as schematically shown in Figure 3. The first step consists in the preparation of an agmatine containing oligomer (pre-polymer) that still carries crosslinkable acrylamide double bonds at the chain ends. This is not isolated and is mixed with the EDA crosslinker just before the deposition by spin coating to achieve *in situ *hydrogel formation. This procedure allows the glass-bound amino groups to take part in the reaction effectively anchoring the hydrogel layer to the glass. During the process of optimisation of the deposition procedure, the monomer concentrations was tuned in order to ensure equal amounts of aminic N-H and acrylamide double bonds. In particular, the acrylamide content was kept constant while varying the EDA and agmatine relative amounts. No samples with less than 50% of EDA were prepared in order to have a polymer material that is stable at least for a week and having good shape retention. When the constructs are soaked in water, and then the hydrogel swells, strong internal stresses are induced in the deposited layer. This leads to a peeling off of the outer layer. However, the part of the film chemically bound to the glass stably rests on the substrate as a thin coating still capable to interact with cells. This nanometric film was characterized by AFM measurements, as below described in detail.

### Optimisation of the crosslinker/agmatine ratio

**Figure 4:**
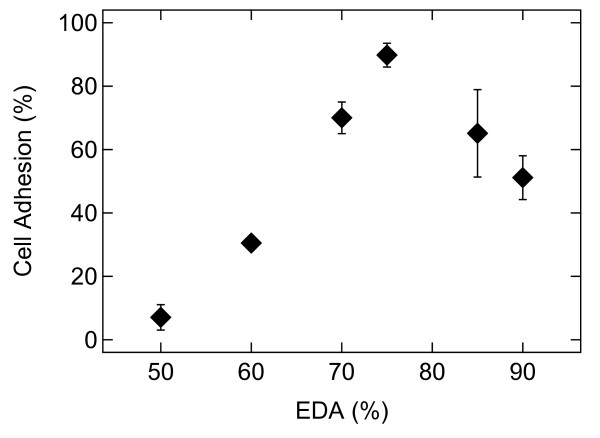
**Cell adhesion versus crosslinker**. Cell adhesion behaviour (normalised against TCPS) versus crosslinker content, given as moles ratio, that is (moles of EDA aminic hydrogens/moles of overall aminic hydrogens) ×100.

Before starting with systematic biological tests, the optimal ratio between crosslinker and agmatine was determined for an effective cell adhesion. A thorough optimization was carried out by analysing a series of AGMA1 hydrogels having variable composition and crosslinking degree and comparing it with a series of ISA23 hydrogels of equal crosslinking degree. A series of gel coated glasses with different composition was prepared and tested for cell adhesion using a single experiment (3 coverslips each) cell adhesion test using MDCK cells at the same conditions reported in the cell culture paragraph. Composition of the samples is reported in Table 1. Results of cell adhesion are reported in Figure 4. The adhesion has a maximum around 75:25 mol/mol crosslinker:agmatine ratio, giving the best balance between adhesion promoter availability. Based on these results, it was decided to concentrate the investigations on the constructs containing this optimal ratio and from now on to call the functionalized hydrogel AGMA1-75 and its analogue ISA23-75.

**Table 1:** Tested AGMA1 hydrogels.

Sample name	BAC (Mol)	EDA (Mol)	Agmatine (Mol)
AGMA1-50	1	0.25	0.50

AGMA1-60	1	0.30	0.40

AGMA1-70	1	0.35	0.30

AGMA1-75	1	0.38	0.25

AGMA1-80	1	0.40	0.20

AGMA1-90	1	0.45	0.10

Composition of the different AGMA1 hydrogels screened in the optimisation procedure.

### Degradation tests of hydrogels

**Figure 5:**
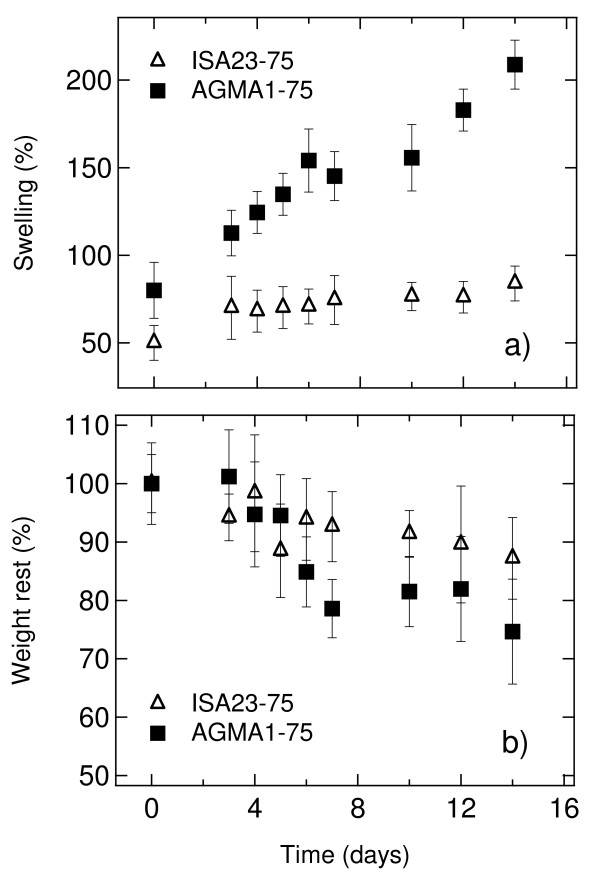
**Degradation kinetics**. Degradation kinetics of AGMA1-75 and ISA23-75 at 37°C in PBS at pH 7.4: a) swelling (%) and b) weight loss (%).

After the determination of the optimal crosslinker/agmatine ratio a series of degradation tests were performed in order to know the behaviour of hydrogels as a function of time. Following the Expressions 2 and 3, the swelling (%) and the weight rest (%) were evaluated for a time interval of 14 days by sampling once a day. Figure 5 shows the degradation kinetics of AGMA1-75 and ISA23-75 in PBS. Figure 5a) shows the swelling of AGMA1-75 which increases almost linearly whereas the swelling of ISA23-75, a part from an initial increase, remains nearly constant. This behaviour is mirrored in the graphs of Figure 5b), where the larger weight loss of AGMA1-75 is apparent. The degradation is then consistent with a gradual breaking of inter-chain linkages. The obtained results indicate that after 14 days the weight of AGMA1-75 is reduced of 25% and that one of ISA23-75 of only 12%.

### Atomic Force Microscopy

**Figure 6:**
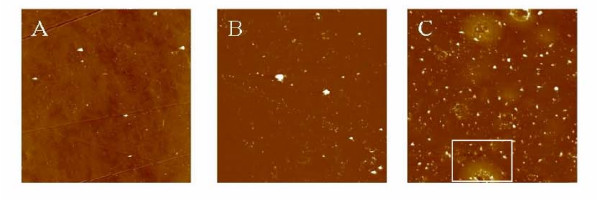
**AFM images**. AFM images of substrates used in the experiment. Horizontal and vertical scale: 50 × 50 *μ*m^2^, 150 nm (A) TCPS. (B) Coverslip surface coated with ISA23-75. (C) Coverslip surface coated with AGMA1-75. The white box highlights a region where the thin hydrogel layer detached from the substrate, assuming a volcano-like appearance.

AFM images reported in Figure 6 show the morphology of different substrates. TCPS surface is uniform and flat except for the presence of grooves a few nanometers deep and many micrometers long. Coverslips coated with hydrogels, instead, are rougher, with granular features ranging from few tens to few hundreds of nanometers in size. AGMA1-75 shows a larger number of grains than ISA23-75, and some volcano-like features. These features originate upon the partial detachment of the hydrogel coating from the substrate. The root-mean-square roughness (the standard deviation of heights around the mean value) of the different substrates was calculated from the AFM images acquired in different locations of the surfaces (averaging on 5–10 images for each sample). Roughness of both ISA23-75 and AGMA1-75 hydrogels was below 20 nm, while that of TCPS was about 11 nm.

### Cell adhesion

**Figure 7:**
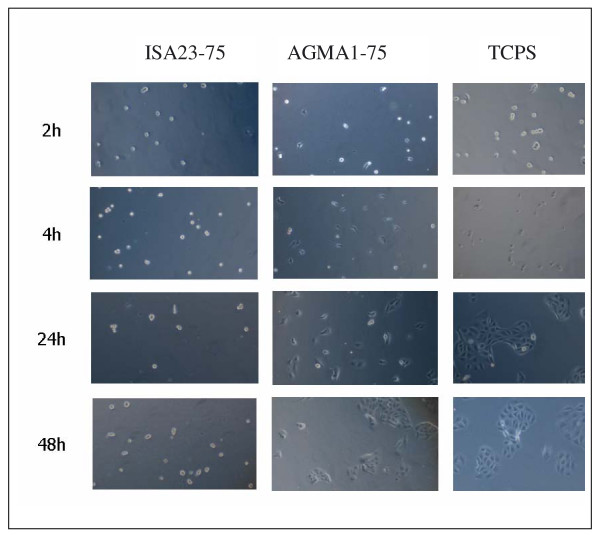
**Optical microscopy images**. Optical microscopy images showing time evolution of MDCK cells on ISA23-75, AGMA1-75 and TCPS. On ISA23-75 the asymmetric cells are few compared to the other substrates. On AGMA1-75 and TCPS the trend is similar, even if on AGMA1-75 the slower vital cell cycle is clearly visible from picture at 24 hr.

**Figure 8:**
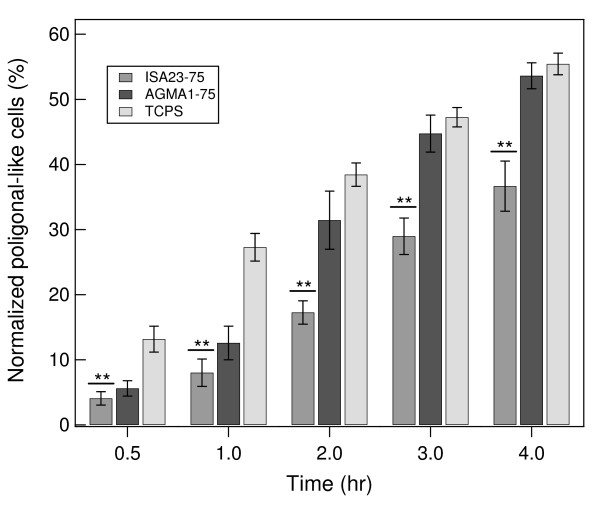
**Cell adhesion versus time on three different substrates**. Cell adhesion versus time on three different substrates: ISA23-75, AGMA1-75 and TCPS. The graph shows the percentage of cells with polygonal-like shape on ISA23-75, AGMA1-75 and TCPS. In the first hour after seeding, MDCK cells have a similar behaviour on ISA23-75 and on AGMA1-75. After a few hours the modified cells are significantly higher on AGMA1-75 than ISA23-75 and approach the percentage on TCPS. Data represent mean ± *σ*. From ANOVA test ** indicates significant at 1% level vs TCPS at the same time.

At first, changes of cell morphology were analyzed during cellular adhesion process and monolayer formation. Figure 7 shows some optical snapshots of cell response on the three investigated substrates at different times, namely 2, 4, 24 and 48 hr. At 2 hr cells on hydrogels and TCPS appear to be mainly round and pearly. After 4 hr a substantial amount of cells on AGMA1-75 and TCPS show an polygonal-like morphology typical of the phenotype of adhered MDCK cells, whereas on ISA23-75 the number of adhered cells is still very low. At 24 and 48 hr after seeding (see Figure 7) cells start to form a monolayer. A certain amount of cells can be also observed on ISA23-75. This effect might be ascribed to the partial absorption of adhesive proteins from serum on the hydrogel. Thus, the modification of the cell morphology becomes manifest within 4 hours after seeding. Figure 8 shows the quantitative evaluation of the percentage of cells presenting the polygonal-like morphology on the different substrates up to 4 hours. Until the first hour the rate of cell modification is higher for TCPS, and afterwards the trend on AGMA1-75 and TCPS is similar. Morphological changes on ISA23-75 are always significantly lower with respect to TCPS.

**Figure 9:**
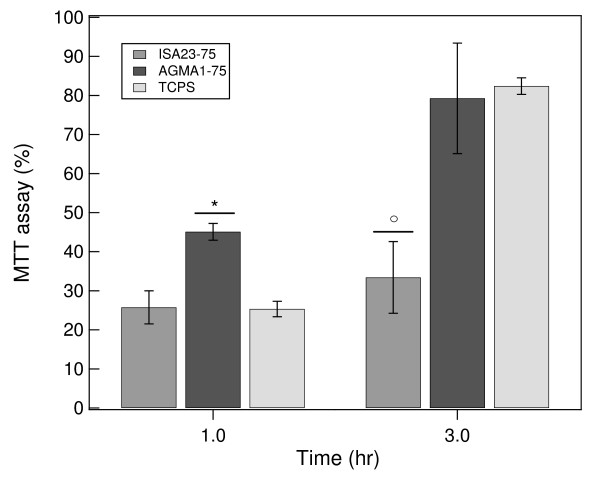
**MTT assay**. MDCK were plated at 4 × 10^4 ^cells/well on TCPS AGMA1-7575 and ISA23-75 hydrogels and cultured for 1 and 3 hours. Viable cells were assessed by MTT assay and MTT-positive cells were expressed as a percentage of adherent cells. Data represent mean ± *σ*. From ANOVA test *P = 0,0399, °P = 0,0444 vs TCPS at the same time.

MTT assay was performed for obtaining a quantitative evaluation of cell viability and adhesion. Figure 9 shows that at 3 hr cells adhesion both on AGMA1-75 and TCPS is comparable. Moreover, at 1 and also at 3 hr after plating MDCK cells show a lower viability on ISA23-75. MTT assay and the study on the morphological profile concordantly indicate that AGMA1-75 promote the cell adhesion.

**Figure 10:**
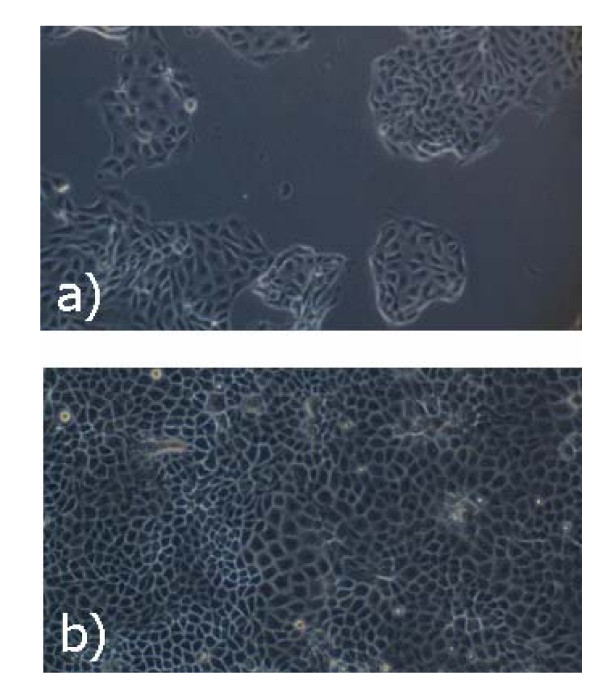
**Optical microscopy images at 72 hr after seeding**. Optical microscopy images showing MDCK on TCPS and AGMA1-75 at 72 hr after seeding. On AGMA1-75 the epithelium shows a large cell clustering: cell spreading of polygonal shape and gray color (a)), whereas on TCPS the cells have reached confluence and start to die (b)).

At longer times cells on TCPS start to proliferate and form clusters. This is the first step to achieve a wide uniform epithelium. On AGMA1-75, instead, cells proliferated slowly. This behavior becomes even more evident at 72 hr (see Figure 10), when cells seeded on TCPS achieve confluence and begin to die. On AGMA1-75, instead, cell clusters are still observed without reaching confluence. Instead MDCK cells grown on ISA23-75 exhibited lower adhesion and slower proliferation compared to AGMA1-75 and TCPS.

In order to evaluate the mechanism of cell adhesion on AGMA1-75, the protein adsorption on hydrogel surface and integrin-binding of agmatine containing hydrogels was investigated. Adhesion experiments with media containing only 0.1% of FBS were carried out. After 4 hr from cell seeding the percentages of adherent cells are: 70.2 (± 2.2) % on TCPS, 47.6 (± 6.5) % on AGMA1-75 and 20.3 (± 3.2) % on ISA23-75. Thus, the cell adhesion is partially due to protein serum adsorption onto AGMA1-75 hydrogel surface.

**Figure 11:**
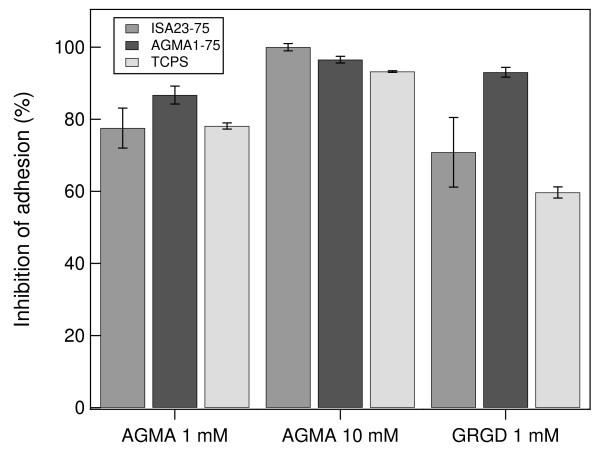
**Cell adhesion inhibition**. The presence in the medium of a soluble polymer bearing the agmatine-BAC sequence in the repeating unit is able to prevent cell adhesion on all the tested substrates. 1 mM GRGD peptide and 1 mM AGMA1 (calculated on repeating unit concentration) have the same effect on cell adhesion inhibition, increasing the soluble polymer concentration to 10 mM does not increase significantly the inhibition of cell adhesion. On ISA23-75 the cells do not adhered in the presence of 10 mM AGMA1 in the medium.

The presence in the medium of a soluble polymer obtained by copolymerization of BAC and agmatine [29] up to a concentration of 1 mM in repeating units, proved to prevent cell adhesion on all the substrates (see Figure 11). Increasing the AGMA1 concentration up to 10 mM did not significantly increase the inhibition of cell adhesion, suggesting that the interested receptors are already almost completely saturated at 1 mM AGMA1. 1 mM GRGD peptide and 1 mM AGMA1 (calculated on repeating unit concentration) have the same effect on cell adhesion inhibition.

### Actin stress fiber and focal adhesion formation on AGMA1-75 hydrogels

**Figure 12:**
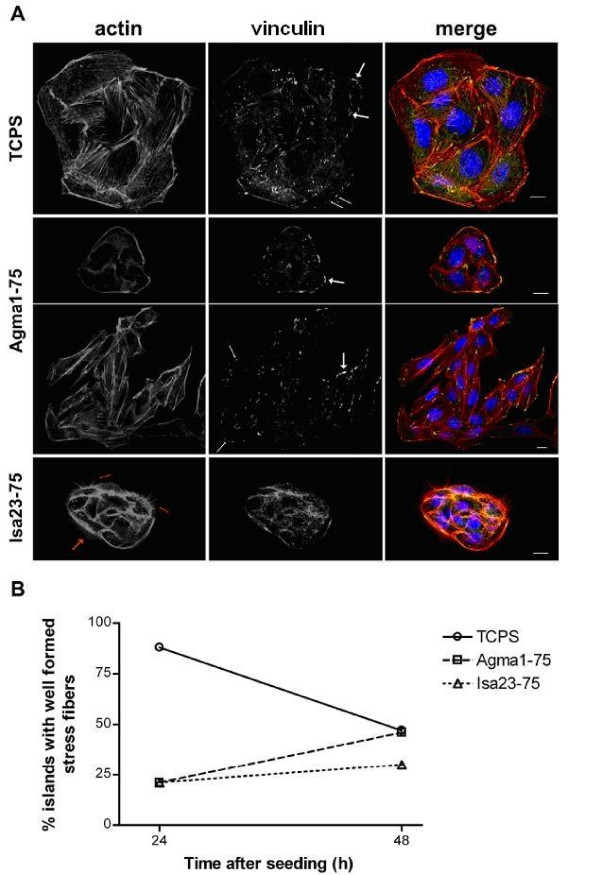
**Immunofluorescence analysis**. Immunofluorescence analysis of actin cytoskeleton and focal contacts of TCPS or hydrogel MDCK growing cells. A) Actin stress fibers (left panel), vinculin stains (middle panel) and their merge (right panel) of MDCK cells 2 days after seeding. Small white arrows indicate short focal contacts and big white arrows indicate the long ones. In the ISA23-75 panels, red small arrows indicate filopodia red big arrows indicate lamellipodia. Nuclei are labeled with DAPI and visualized in blue. Scale bar: 10 *μ*m. B) The percentage of cell islands with well formed stress fibers is plotted against the time after seeding. 62 (TCPS), 105 (AGMA1-75) and 72 (ISA23-75) cell islands from 3 different experiments were scored.

RGD sequence from fibronectin has been shown to interact with *α*_V_*β*_3 _integrin [12] that is expressed on MDCK cells [30]. Integrin occupancy and clustering determine the activation of a signaling pathway that ultimately affects cell adhesion, spreading and consequently cell migration, by the interaction with cytoskele-tal proteins [34,35]. Though the cell adhesion on TCPS and AGMA1-75 4 hr after seeding is similar, cell spreading is less effective on the AGMA1-75 hydrogel compared to TCPS (50% ± 14% of spread cells on AGMA1-75 and 96% ± 1% of spread cells on TCPS, n = 10). In order to determine whether AGMA1-75 hydrogels affect cytoskeleton and focal adhesion organization of MDCK cells, actin filaments and vinculin, an adhesion component, were visualized. It was found that TCPS growing cells, 24 hr after seeding, present 88% (*n *= 24) of cell islands with well formed stress fibers and this value decreased 48 hr after seeding to 47% (*n *= 38). AGMA1-75 and ISA23-75 growing cells had the opposite behavior: the number of stress fibers containing islands increased during time with a steeper increase for AGMA1-75 compare to ISA23-75 (AGMA1-75 = 21%, *n *= 34 and 46%, *n *= 71; ISA23-75 = 21%, *n *= 19 and 30%, *n *= 53, 24 hr and 48 hr after seeding, respectively, as shown in Figure 12B). TCPS and AGMA1-75 growing cells present heterogeneity in terms of length, size and thickness of vinculin-stained focal adhesion (Figure 12A). On both substrates it is possible to find short (small arrows) or long (big arrows) focal adhesions 48 hr after seeding.

## Discussion

The hydrogels chosen for the experiments, ISA23 and AGMA1, were subjected to different preliminary tests of characterization. At first it was investigated the swelling of hydrogels in different fluids, namely water, PBS and ethanol. As shown in Figure 3, for the same amount of crosslinker, both ISA23 and AGMA1 show higher water and PBS absorption with respect to ethanol. This behaviour is expected owing to the hydrophilic structure of the hydrogels under investigation. Consequently hydrogels, swelled in water and PBS, appear to be softer with respect to the corresponding hydrogels swelled in ethanol. With the main purpose of making hydrogels easily of handling and suitable for optical measurements, a procedure for anchoring the polymers to the glass substrate was developed. In order to extend the stability of hydrogels it was never used less crosslinker than 50%. For both ISA23 and AGMA1, the hydrogel layer attached to the glass after the swelling and peeling operations present a roughness larger (about 20 nm) than that one of TCPS surface (about 11 nm), as shown by AFM measurements. With this technique it was also proved that the morphology of the supported hydrogels is stable on the time scale of several days upon exposure to ambient conditions, as well as upon wetting-dewetting cycle. Since during this period hydrogels preserve their features, it is assured the feasibility of performing cell culture experiments in an appropriate timescale. The crosslinker/agmatine ratio for the maximal MDCK cell adhesion was assessed to be 75:25 mol/mol crosslinker: agmatine ratio (see Figure 4). The corresponding hydrogels, named AGMA1-75 and ISA23-75 were subjected to a series of degradation tests. It was observed that after 14 days the swelling (%) of AGMA1-75 doubles with respect to the first day, whereas the swelling (%) of ISA23-75 remains more less invariant (see Figure 5a)). The weight loss (%) reflects this behaviour where AGMA1-75 loses much more weight with respect to ISA23-75 (see Figure 5b)). The measured trends confirm the expected larger stiffening of the non-functionalized hydrogels. These experimental data reveal that both types of hydrogels do not widely degrade under the reported conditions over the considered time interval. However, even if the experiments were carried out in an environment similar to that of biological fluids, it should be considered that degradation experiments performed in vivo might provide different results. As reported in the work of Ferruti et al. [33], the degradation products, deriving from the hydrolytic scission of amidic functions placed in the repeating units of PAAs, are fully non-toxic. In these experiments no evidence of cytotoxicity came out.

After the described procedures for the preparation of hydrogels substrates and their characterization, a series of tests on cell adhesion and proliferation were carried out. MDCK were plated on the two types of hydrogels and on TCPS. Within 1 hr after plating no evident differences can be observed between AGMA1-75 and ISA23-75 and the amount of adhered cells on these substrates is significant lower than on TCPS (see Figure 8). After 3 hr the trend is substantially different and the adhesion on AGMA1-75 is comparable to that on TCPS (within one standard deviation), whereas on ISA23-75 remains definitely lower (see Figures 8 and 9). After 1-2 days effective MDCK proliferation on TCPS can be observed whereas this process on AGMA1-75 appears to be slowed down (see Figure 7). After 3 days, meanwhile the cells on TCPS achieve confluence, the cells on AGMA1-75 form clusters and no confluence is observed (see Figure 10). This effect may be explained considering that, despite the fact that PAA hydrogel layer were supported by a rigid material, cells probably experienced a more compliant substrate than TCPS. It is generally recognized that focal contact formation and cytoskeletal assembly may be partially hindered on hydrogel surface with a subsequent slowing down cell growth [36].

In order to assert that the major effectiveness of cell adhesion on AGMA1-75 is due to its functionalisation, soluble AGMA1 was dissolved in the medium with the purpose of verifying inhibition of adhesion on the three different substrates, namely AGMA1-75, ISA23-75 and TCPS. The results confirm that AGMA1 prevent the cell adhesion at comparable amount of soluble GRGD. Interestingly it can be also observed that the maximal inhibition is obtained on ISA23-75, whereas a small amount of adhesion persists on TCPS and AGMA1-75, confirming the low aptness of non-functionalized hydrogels to cell adhesion (see Figure 11). Since GRGD and AGMA1 produce comparable inhibition, it can be reasonably concluded that they both compounds bind the same integrin receptor (i. e. *α*_V_*β*_3 _expressed on MDCK cell [12,30]).

Optical images indicate that the cells adhered on AGMA1-75 are less spread with respect on TCPS. This behavior could be ascribed, as mentioned above, to the occurrence of opposite stimuli to the cells: the compliance of the hydrogel surface, that can prevent a strong cell-substratum interaction and stress fiber formation, and the presence of integrin ligands, that favors a more effective cell substratum adhesion. The behaviour of the actin stress fibers on the different substrates is shown in Figure 12B). Slower actin stress fiber formation on AGMA1-75 and ISA23-75 matched the slower spreading on these hydrogels. Chemical properties can act together with physical characteristics of substrates to affect cell adhesion. It was shown that the presentation of integrin ligands in a clusterized form enables the formation of focal contacts and stress fibers [37] and determines cell spreading [38]. A uniform low density of integrin ligands, instead, is unable to support stress fiber formation [37]. Therefore, slower spreading and stress fiber formation on AGMA1-75 hydrogels could be also due to a more uniform (i. e. not clusterized) presentation of integrin ligands compare to the TCPS. It is also interesting to note that on ISA23-75 cell islands often showed membrane structures as filopodia and lamellipodia (Figure 12A, red arrows) indicating not stable focal contacts and a tendency to cell migration [39].

## Conclusion

A robust protocol for the production of supported nanometric hydrogel layers on transparent substrates for cell culture was developed. It was relied on the versatility of poly(amidoamine) hydrogels, that were engineered from a simple amphoteric biocompatible system (ISA23-75) to incorporate RGD-mimicking units (AGMA1-75) by introducing guanidine pendants and optimising their amount in order to optimize cell adhesion. Anchored nanosized hydrogel layers were obtained by in situ polymerization carried out on glass substrates purposely modified with – aminopropyltriethoxysilane, followed by swelling in water, which invariably lead to spontaneous delamination of the external bulk of the hydrogel. The resultant hydrogel layers showed flexible hydrated surface, optically transparent and free of defects causing visible light scattering. It may be therefore assumed that they may open the way to devices suitable for optical high-resolution microscopy. AGMA1 hydrogel layers exhibited towards epithelial cells (MDCK) a level of cell adhesion comparable to that of commercial plastic substrates for tissue culturing even in the presence of only 0.1% of FBS. On the contrary, a plain amphoteric PAA, ISA23-75, showed a vastly inferior cell adhesion, thus demonstrating that epithelial cell adhesion is not a general property of amphoteric PAAs. Integrins are the major receptors for the extracellular matrix in MDCK cells and they affect epithelial cell polarization. AGMA1-75 substrate, even if it guarantees a good cell adhesion, seems to delay the appearance of a polarized epithelium, making the spreading and the proliferation slower than on TCPS. This effect could be interesting if transposed to stem cells, which need an environment able to maintain them in a quiescent state to keep their self-renewal and multi-potentiality until they start differentiation to supply new cells for the normal tissue turn over [40]. AGMA1 hydrogels, as PAAs in general [41-43], may undergo further physical and chemical modifications to meet specific requirements. Moreover the adopted synthetic process is simple and easily scaleable. As final conclusion, supported functionalized hydrogels are very promising substrate for cell culture in biomedical applications.

## Competing interests

The authors declare that they have no competing interests.

## Authors' contributions

EJ carried out the cell culture experiments. EE prepared hydrogels. SR performed confocal microscopy imaging. MI and AP performed AFM measurements. CL, ER, PF and PM participated in the design of the study and coordination. All authors contributed in the preparation of the manuscript.
